# Does the severity of pain correlate with severity of functional disability? Factors influencing ‘patient reported outcome measures’ in spinal patients

**DOI:** 10.1051/sicotj/2018029

**Published:** 2018-10-01

**Authors:** Andrew P. MacCormick, Himanshu Sharma

**Affiliations:** 1 Plymouth University Peninsula School of Medicine Dentistry, Plymouth UK; 2 Department of Surgery, Plymouth Hospitals NHS Trust, Derriford Hospital, Plymouth PL6 8DH UK

**Keywords:** Patient reported outcome measures, PROMs, Oswestry disability index, VAS pain score, Lumbar spinal surgery

## Abstract

*Aims*: To assess correlation between the Visual Analogue Scale (VAS) pain score and the Oswestry Disability Index (ODI) and which patient factors can influence patient-reported outcome measures (PROMs). This study also aims to assess the response to the sexual function question of the ODI.

*Methods*: Retrospective analysis of 200 consecutive patients undergoing a range of different lumbar spinal procedures between July 2012 and September 2015 was performed. Subgroup analysis was also performed on the 122 patients who underwent microdiscectomy and/or decompression procedures only. Data from notes and clinical letters from the patient's first clinic appointment were collected. In addition to these outcome measures, data were also extracted regarding patients' gender, age, smoking status, alcohol use, employment and mental health status.

*Results*: Significant correlation was found between VAS pain score and ODI (*p* = 0.002) and between VAS pain score and question 1 of ODI (*p* = 0.0001). A lower ODI score was reported at time of surgery by those in employment compared to those who are unemployed (*p* = 0.008). In addition to this, a lower ODI score was reported in those who are self-employed compared to those in employment (*p* = 0.048) in both cohorts. A significantly higher mean ODI score was shown within the subgroup analysis for current smokers (*p* = 0.02). None of the other patient factors that were analysed were found to affect PROMs. 65% of patients answered the sexual function question of the ODI.

*Conclusions*: Significant correlation was demonstrated between VAS pain score and ODI. Those who are in employment are far more likely to report a lower ODI score than those who are unemployed at the time of surgery. Self-employed patients were found to have reported a significantly lower ODI score than those who are in employment. Smoking cessation should be encouraged as those who are current smokers may be more likely to report a higher ODI. As 65% of patients decided to answer the sexual function question of the ODI, this supports its further use.

## Background

Back pain has been defined as the leading cause of disability in the UK [1], affecting 15% of adults and the impact that this can have on a patient's quality of life (QOL) can be severe. Physicians are now encouraged to monitor the impact of a patient's condition on their everyday living both before and after surgery [3].

**Figure 1:**
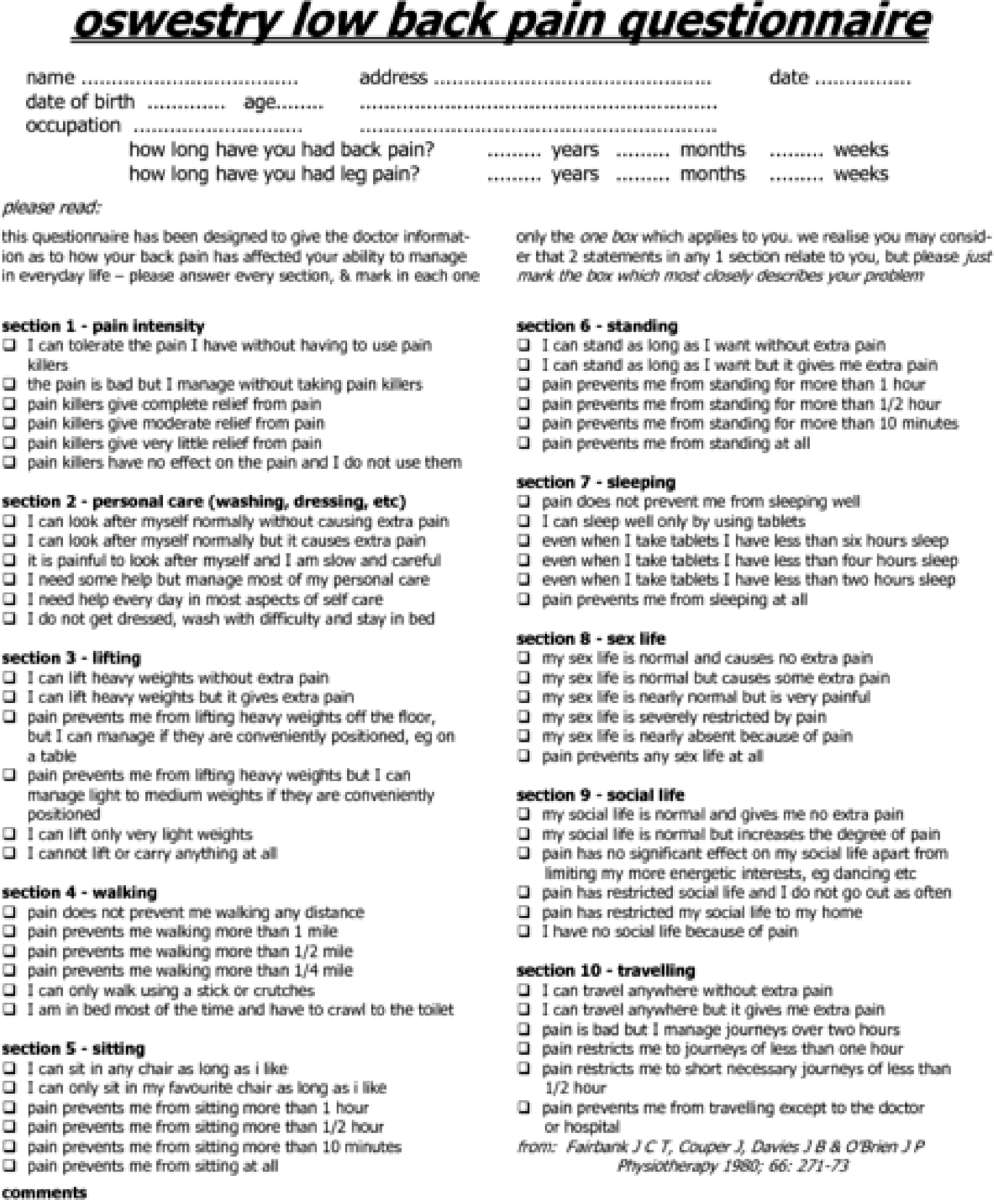
Summary of the ODI.

The Visual Analogue Scale (VAS) and the Oswestry Disability Index (ODI) are two commonly used systems to measure patient-reported outcome measures (PROMs) in spinal patients. The VAS is a unidimensional measure of pain intensity that is widely used for a range of conditions. The ODI, on the other hand, is a condition-specific measure for the assessment of outcomes in spinal pathologies. This tool, developed by Fairbank [4] in 1980, has been extensively tested by various authors for its applicability and reliability [5]. They concluded that it is an effective instrument for the disability assessment of lumbar spine pathology as it addresses both pain and function (Figure 1).

A range of different patient factors have been shown to also impact these outcome measures. A large proportion of patients who are off work due to their back pain have been found to remain off work even after surgery [6], so research is required to assess how the highest level of HRQoL can be achieved. The first step for this process will be to assess which patient groups to target this at, and hence why this study will aim to assess which patient factors can influence PROMs.

## Aims

The aims of this study are to assess correlation between the VAS pain score and the ODI and which patient factors can influence PROMs. This study will also assess the response to the sexual function question of the ODI.

## Methods

### Patients

The setting for this study was a multicultural tertiary neurosurgery centre in a developed western country. Retrospective analysis was undertaken on 200 consecutive patients who underwent a range of different lumbar spinal procedures between July 2012 and September 2015. This patient group was defined as cohort 1. Subgroup analysis for patient factors was also performed on the 122 patients within this cohort who underwent microdiscectomy and/or decompression procedures and this patient group was defined as cohort 2. Inclusion criteria were that the patients were all seen within this time period and were all under the care of and operated on by the same surgeon to ensure consistency of data. All patients with incomplete information regarding their VAS pain score or ODI score were excluded from this analysis and the subsequent data interpretation. When statistical analysis was required, patients were divided into VAS groups based on their level of pain: mild (<4), moderate (5–7) and severe (>8).

### Treatment

All of the patients in the main cohort group underwent a range of different lumbar spinal procedures including microdiscectomy, decompression, fusion, nerve root injection and laminectomy. The patients in the subgroup only underwent microdiscectomy and decompression procedures. These operations were performed by the same surgeon who saw them at their first clinic consultation.

### Outcome measure and baseline data

Data were collected using the notes and clinical letters from the patient's first clinic appointment. PROMs were all taken at this appointment including ODI and VAS pain score. The ODI index, which includes 10 items, is a commonly used outcome measure for reporting how a patient's back or leg pain is affecting their ability to manage daily living. Recent literature has demonstrated that it shows good psychometric properties and is an effective tool for reporting functional outcome following spinal surgery [7]. Each of the 10 items on the index is scored from 0 to 5, with the maximum possible score being 50 [8]. To achieve a percentage, as is reported in this study, the total score must be multiplied by 2. Patients were not grouped on the basis of their ODI score. The VAS pain scale is one of the most commonly used methods to record patient pain [10]. Patients were asked to report their current level of back and leg pain on a scale of 0–10, representing a scale of pain from none at all to the most excruciating pain possible.

In addition to these outcome measures, data were also extracted regarding patients' gender, age, smoking status, alcohol use, employment and mental health status. This was also obtained from the notes and clinical letters from patients' first clinic appointment.

### Statistical analysis

Statistical analysis was performed using the SPSS statistical software [10]. VAS pain score and ODI score were analysed using a two-sample (unpaired) t-test to analyse the difference between the means. Mean ODI score was compared with mean VAS pain score for each of the mild, moderate and severe groups.

When considering the other patient factors, the same statistical analysis software and method was used. The divisions within each of these groups are demonstrated in Table 1. A *p* value for all data of <0.05 was considered significant.

**Table 1:**
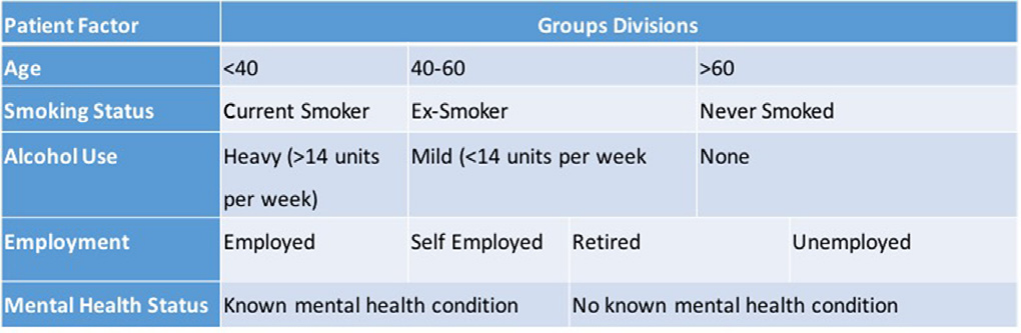
Divisions within each patient factor group.

## Results – main cohort

### Correlation between VAS pain score and ODI

**Figure 2:**
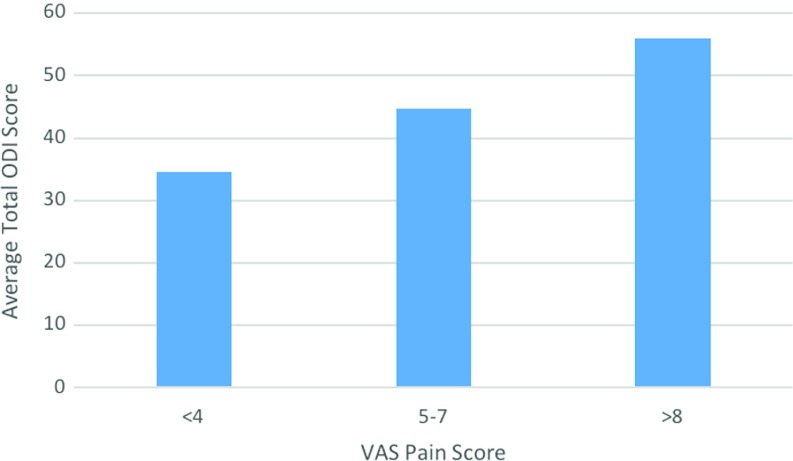
Graph comparing VAS pain score with average total ODI score.

Results from all 200 patients were obtained. Significant correlation was found in all groups of patients. When comparing the mild VAS pain score group to the moderate group, a difference in mean overall ODI score of 10.2 was found (*p* = 0.002 (95% CI 3.83-16.58)). A similar difference was also seen when comparing the moderate group to the severe group, when an average ODI score difference of 11.19 was found (*p* ≤ 0.0001 (95% CI 6.28–16.10)) (Figure 2).

**Figure 3:**
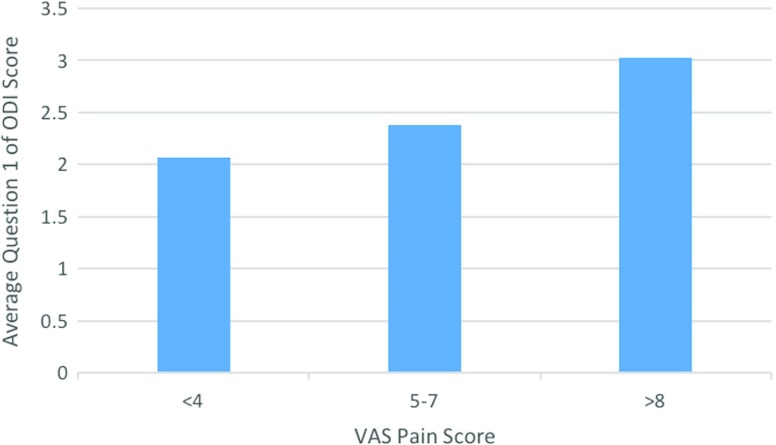
Graph comparing VAS pain score with average question 1 score of the ODI.

Statistical significance was also found when comparing VAS pain score to question 1 of the ODI, that being the question focusing on the severity of current pain levels. Comparing the moderate group to the severe group, there was an average question 1 VAS pain score difference of 0.65 (*p* = 0.0001 (95% CI 0.33–0.96)) (Figure 3).

### Impact of patient factors on outcome measures

**Figure 4:**
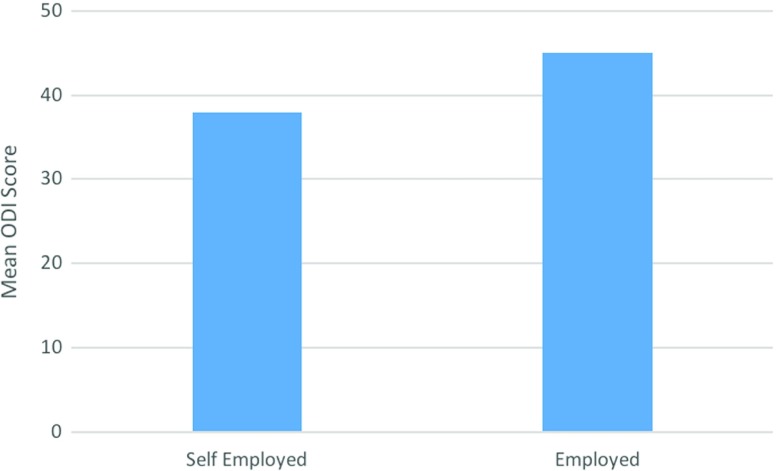
Graphical representation of the mean ODI score of those that are self-employed compared to those in employment.

Employment status was found to have the most significant impact on PROMs. A significantly lower mean ODI score was found when comparing those who are self-employed (*n* = 22) to those who are in public or private sector employment (*n* = 46). An ODI difference of 6.8 (*p* = 0.0479 (95% CI 0.06–13.57)) was observed (Figure 4).

**Figure 5:**
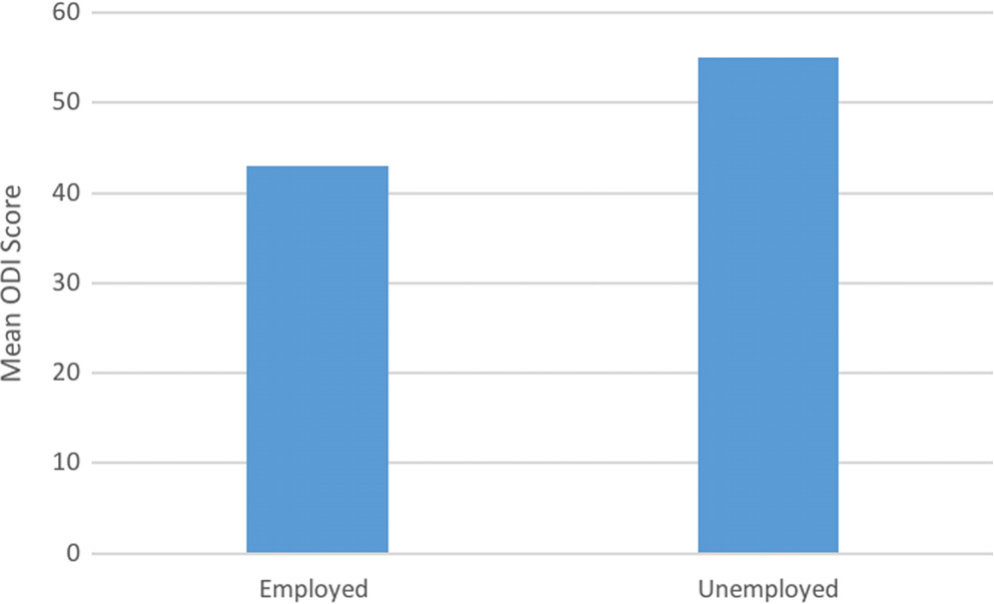
Graphical representation of the mean ODI score of those that are employed compared to those that are unemployed.

Furthermore, more significant results were seen when comparing those who are currently in any form of employment (*n* = 68) to those who are unemployed (*n* = 29). An ODI difference of 12.2 (*p* = 0.0078 (95% CI 3.24–20.76)) was found, with those who are in employment reporting a lower ODI score (Figure 5).

When considering the other patient factors that were analysed: age, gender, smoking status and alcohol use, no other statistically significant differences were found within these groups. The results that were obtained are shown in Table 2.

**Table 2:**
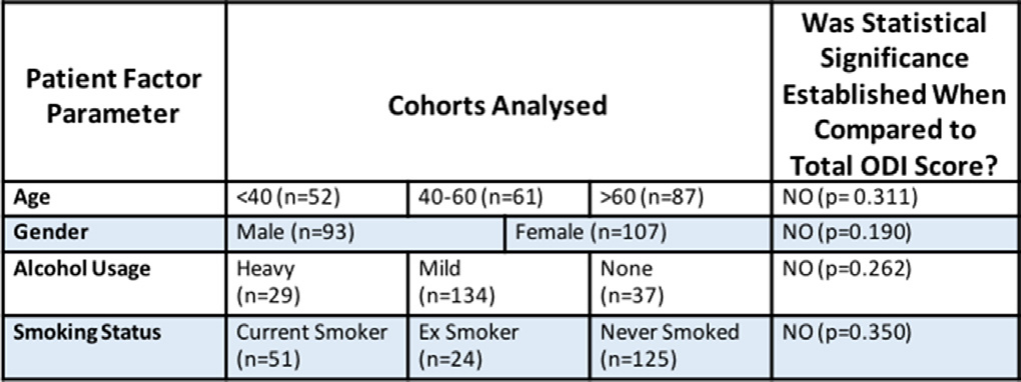
Summary of the results of the impact of the other measured patient factors on ODI within Cohort 1.

### Response to question 8 (sexual function) of the ODI

**Figure 6:**
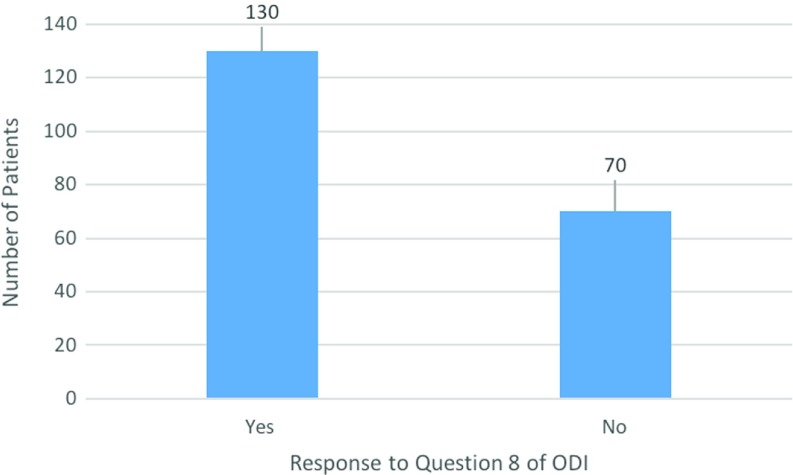
Graphical representation of the response to question 8 of the ODI.

Of the 200 patients, 130 patients (65%) chose to answer question 8, regarding sexual function, of the ODI while 70 patients (35%) chose not to. In the patients who declined to answer this question, this had no bearing on their responses to the rest of the questions as a completion rate of 100% was achieved for the questionnaire (Figure 6).

## Results – subgroup analysis

### Impact of patient factors on outcome measures

The results from the subgroup analysis of cohort 2 also demonstrated a significantly lower mean ODI score in those who are self-employed (*n* = 15) compared to those who are in any public or private sector employment (*n* = 31). There was a mean ODI score difference of 7.2 (*p* = 0.032 (95% CI 0.91–13.32)) with those who are self-employed reporting the lower scores.

Furthermore, similarly significant results were also found when comparing those who are currently in any form of employment, including self-employed, (*n* = 46) to those who are unemployed (*n* = 29). A mean ODI score difference of 11.2 (*p* = 0.0092 (95% CI 2.98–18.76)) was demonstrated.

Interestingly, within this subgroup analysis, a significant difference was found when comparing those who currently smoke with those who have never smoked. A mean ODI score difference of 7.9 (*p* = 0.02 (95% CI 1.12–14.76)) was shown with those who have never smoked demonstrating the lower mean ODI score.

As with cohort 1, no statistically significant differences were found when analyzing age, gender and alcohol use.

## Discussion

The significant correlation between the VAS pain score and the ODI demonstrated in this study supports their simultaneous use to enable both corroboration of score and further understanding of different sources of pain. The main conclusion from a recent meta-analysis found ‘little correlation between the VAS pain score and ODI’, so extensive further research was recommended in order to assess the correlation between these two measures further [11]. The results from this study aim to do this by clearly demonstrating the positive correlation between these two measures, thus further supporting the need for further research into this topic.

Due to the high percentage of those with back pain not returning to work after surgery, further consideration into the reasons behind this is essential. Recent studies have highlighted the need for research to occur into the impact of employment status on PROMs [13], and this was in keeping with this studies finding of employment status being the only patient factor to do this. Those in employment were shown to be more likely to report a lower ODI score than those who are currently unemployed. While assumptions that those who are unemployed may be somewhat exaggerating, the degree of their disability cannot be made from this study alone, this must be considered. Along with this, findings from this study that those who are self-employed were more likely to report a lower ODI score than those in employment is in line with recent literature. This demonstrated that those who are self-employed are likely to be more determined to return to work quicker than those who are employed by a company due to their lack of entitled sick pay [14]. This is an important consideration when assessing the reasons why some patients fail to return to work after surgery.

The subgroup analysis of the microdiscectomy and decompression patient cohort demonstrated a significantly greater mean ODI score in those that currently smoke. Recent literature has demonstrated increased complication rates in smokers [15], so this adds further evidence to the importance of counselling smokers with regard to cessation prior to their procedure as this could also impact the long-term outcomes after their operation.

Significant attention has also been given to question 8 of the ODI in recent research, providing conflicting information. Response rates of 18% [16], 36% [17] and 52% [18] have been found for this question on three recent large studies. The response rate of 65% found in this study is higher than the other pieces of literature; this, however, still does show that there is a degree of apprehension from patients regarding the completion of this question.

## Limitations

The greatest limitation within this study is the inclusion of a range of different lumbar spinal procedures in the main cohort of patients which ranged from nerve root blocks to spinal fusions. As a result of this, the subgroup analysis was performed on the biggest patient population that shared the same operative procedure. The patient cohort size for this study was moderate but was strengthened due to the single surgeon experience that was involved in the analysis in order to maintain consistency of data.

## Conclusion

This study has demonstrated significant correlation between the VAS pain score and the ODI. This serves to support their simultaneous use in clinical practice. In addition to this, employment status and smoking status have been shown to have a significant impact on PROMs. This should be taken into consideration in clinical practice as additional measures could be put in place in order to try and encourage eventual return to employment and smoking cessation. A response rate of the sexual function question of the ODI of 65% is considered adequate and this encourages its continued use.

## Conflict of interest

The authors declare that they have no conflict of interest.
